# Front-Loading Sputum Microscopy Services: An Opportunity to Optimise Smear-Based Case Detection of Tuberculosis in High Prevalence Countries

**DOI:** 10.1155/2009/398767

**Published:** 2009-03-15

**Authors:** Andy Ramsay, Mohammed Ahmed Yassin, Alexis Cambanis, Susumu Hirao, Ahmad Almotawa, Mohamed Gammo, Lovett Lawson, Izabel Arbide, Nasher Al-Aghbari, Najla Al-Sonboli, Jeevan Bahadur Sherchand, Punita Gauchan, Luis Eduardo Cuevas

**Affiliations:** ^1^Liverpool School of Tropical Medicine, Liverpool L3 5QA, UK; ^2^UNICEF/UNDP/World Bank/WHO Special Programme for Research and Training in Tropical Diseases, World Health Organization, 1211 Geneva 27, Switzerland; ^3^TB and Leprosy Control Programme, Southern Region Health Bureau, P.O. Box 149, Awassa, Ethiopia; ^4^Zankli Medical Centre, P.O. Box 7745, Abuja, Nigeria; ^5^Bushullo Major Health Centre, P.O. Box 5, Awassa, Ethiopia; ^6^National Tuberculosis Institute, Sana'a, Yemen; ^7^Microbiology and Parasitology and Health Research Laboratory, Infectious and Tropical Disease Research Centre, Institute of Medicine, Tribhuvan University, Kathmandu, Nepal P.O. Box 10404, Nepal

## Abstract

*Setting*. Ethiopia, Nepal, Nigeria, and Yemen. *Objective*. To reduce the time to complete sputum microscopy. *Design*. Cross-sectional surveys enrolling 923 patients with chronic cough in the 4 countries and using similar protocols. Spot-morning-spot sputum specimens were collected. An additional sputum specimen (Xspot) was collected one hour after the first, and the yields of the first two or the three specimens collected as spot-morning-spot or spot-Xspot-morning were compared. *Results*. 216 patients had ≥ one positive smear. 210 (97%) were identified by the spot-morning-spot, and 210 (97%) were identified by the spot-Xspot-morning specimens, with 203 and 200 identified by the first 2 specimens of each approach, respectively. Neither difference was significant. *Conclusions*. The time to complete smear microscopy could be reduced.

## 1. Introduction

New diagnostics for pulmonary tuberculosis (PTB) that are more sensitive than sputum smear microscopy and suitable for primary health care (PHC) services in low- and middle-income countries (LMICs) are urgently needed. Although several promising new diagnostics are under development, they are unlikely to become widely available at the lower levels of LMIC health services in the near future [1]. In the meantime, TB case detection must be improved through the optimal use of existing diagnostic tools. The optimisation of sputum microscopy services, often the only TB diagnostic services possible at PHC level in LMICs, is urgently needed [2–6]. Smear microscopy has several limitations, including poor sensitivity, being labour intensive, and requiring skilled microscopists. Furthermore, the need to collect serial sputum specimens over multiple patient visits results in a protracted diagnostic process with high rates of patient drop-out [7, 8].

Recent studies examining the yield of serial sputum specimens, usually collected as *spot-morning-spot*, have reported that the majority of patients with smear-positive PTB are identified by the first two sputum specimens [2], and the World Health Organization (WHO) has recently changed its policy in this respect, reducing the minimum number of sputum specimens examined for each patient from three to two [9]. This will result in reduced laboratory workloads in many settings, with the potential of improving the quality of sputum microscopy [10]. Case detection may thus be expected to increase in locations where the number of new cases detected through improved microscopy quality exceeds the 2% to 5% of cases missed by not examining the third specimen [5].

The policy changes do not, however, specify the timing for the collection of the two specimens. If specimens were collected at the time of consultation (*1st* *on-the-spot*) and the morning of the following day (*morning* sample), the *spot-morning* specimens would still require a minimum of two visits, which is the minimum required by the *spot-morning-spot* scheme currently used in most diagnostic centres of LMICs. In addition, the *spot-morning* and *spot-morning-spot* schemes still examine a substantial proportion of samples the second day of the diagnostic process. If the process could be “front-loaded”, that is, if all or the majority of sputum collections were conducted the first day of the diagnostic process, this may reduce the number of visits required and reduce patient drop-out, particularly if results could be made available the same day.

This study describes the yield of a front-loaded diagnostic scheme, in which an additional *on-the-spot* specimen (referred here as the *Xspot*) is collected one hour after the first spot specimen. We hypothesised that the yield of this specimen is similar to the yield of other specimens collected on the spot, and that the overall yields of the *spot-Xspot-morning* and the standard *spot-morning-spot* schemes are similar. In addition, the study explores whether the first two specimens collected identify the majority of smear-positive patients.

## 2. Materials and Methods

**Figure 1:**
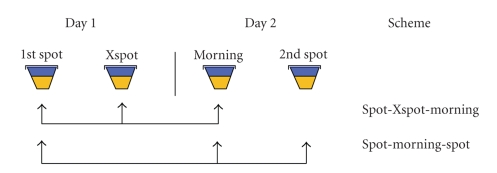
Sputum samples collected and analysed for each scheme.

Four separate studies were conducted in Ethiopia, Nepal, Nigeria, and Yemen using similar study designs. All individuals 15 years of age or older with cough for ≥3 weeks were invited to participate and were enrolled consecutively at the time of presentation to the health services and after obtaining informed consent. The services in all four settings were busy outpatient clinics of district hospitals that were integrated with the National TB Control Programme of the country. Individuals either self-presented to these clinics or had been referred from peripheral health centres for assessment. Patients were requested to submit 3 sputum specimens as *spot-morning-spot* (the standard approach), and an additional specimen was collected one hour after the first (the *Xspot*) on the first day of consultation (Figure 1). All smears were stained using the hot Ziehl-Neelsen method, were read blindly by trained laboratory technicians, and graded using the WHO/IUATLD system. In accordance with the recent WHO policy changes, all smears with ≥1 acid fast bacillus /100 high power fields were considered positive. The main difference in study design between the four sites was that all *morning* specimens collected in Nepal and Yemen were cultured on solid egg-based media to allow for the calculation of sensitivity and specificity when considering culture as the reference standard. The four study sites had internal quality control procedures in place. External quality assessment (EQA) of smear microscopy was conducted by the Liverpool School of Tropical Medicine (LSTM), UK. For all 4 sites, there was >98% agreement between the results of the study microscopists and those of the controllers at LSTM.

Data were analysed to describe the yield of single smears and the cumulative yields of the standard (*spot-morning-spot*) versus the front-loaded (*spot-Xspot-morning*) schemes and of the two smear *spot-morning* versus the front-loaded *spot-Xspot* schemes. Proportions were described as percentages and 95% confidence intervals (95%CI). A number of patients (62, 7%) did not have complete sets of sputum specimens for analysis. These patients were included in the analysis for the additional yield, and missing smears were considered as negative. As the schemes were not independent, comparisons of marginal proportions were made using matched McNemar tests. These included comparisons of the extra yield of the third sputum for each scheme; the yield of the “two” sample schemes and the comparison of the dropouts if patients were examined by the standard and frontloaded schemes. The extra yield of the third sputum for each scheme was summarised in 3 × 3 tables using the categories “positive on at least one of the first two samples”, “positive on the third sample only”, and “negative on all samples” and stratified by study setting. As this analysis required complete sets of data, the 62 (7%) patients with missing sputum samples were excluded from this analysis. The yield for the “two” sample schemes was also compared with 2 × 2 tables with the indicators for each scheme being “positive on at least one of the two specimens” and “negative on both specimens”. Patients with one of the two specimens missing (36, 4%) were classified as negative if the specimen available was negative. McNemar and Stuart MaxWell tests for marginal heterogeneity were used for comparison of the two and three specimen schemes, respectively, stratified by study site.

Partial results of the studies in Ethiopia and Nigeria, which described the yield of two smears collected as *spot* and *Xspot* in a single day, have been reported previously [3, 4]. However, this report includes the yields of the *spot-morning-spot* and *spot-Xspot-morning* schemes from these sites as well as the data from two additional sites conducting studies with similar design approaches.

Ethical approval for the study protocols was obtained from the research ethics committees of the Liverpool School of Tropical Medicine and the Institutional Review Boards of the participating institutions in Ethiopia, Nepal, Nigeria, and Yemen.

## 3. Results

A total of 923 consecutive patients were recruited. Of these, 243 were enrolled in Ethiopia, 206 in Nepal, 224 in Nigeria, and 250 in Yemen. Two hundred and sixteen (23%) of the 923 patients had one or more positive smears. The *first-spot*, *Xspot*, *morning,* and *second-spot* specimens were graded positive in 181 (20%), 186 (20%), 185 (20%), and 176 (19%) patients, respectively, as shown in Table 1. In addition to the 181 patients identified by the first*-spot*, 22 (10%) and 7 (3%) additional patients were detected by the *morning* and second-*spot* specimens, respectively, resulting in a cumulative yield for the *spot-morning-spot* scheme of 210 (23%) patients. In comparison, the *Xspot* and *morning* specimens of the front-loaded approach detected 18 (8%) and 11 (5%) additional patients, also resulting in a cumulative yield for the *spot-Xspot-morning* scheme of 210 (23%) patients. The yield of the third smear was the same in both approaches, independently on whether this was thesecond *spot* or the *morning* specimen (*P* > .5). A comparison of the yield of the third smear for samples collected using the standard (*spot-morning-spot*) and the front-loading (*spot-Xspot-morning*) approaches by country is shown in Table 2. Although there were a few discrepant results in the yield of the third specimen across the schemes, the direction of these discrepancies varied across study sites, and the discrepancies in each study site or combined were not statistically significant.

The first two (*spot-morning*) smears of the *spot-morning-spot* approach identified 203 (97%) smear-positive patients. These two smears, therefore, would have missed 7 (3%, 95%CI 1%–6%) of the cases identified by the three smears, as shown in Table 1. The first two (*spot-Xspot*) smears of the *spot-spot-morning* approach identified 199 (95%, 91%–97%) smear-positive patients. These two spot smears, therefore, would have missed 11 cases (5%, 95%CI 3%–9%) identified by the three smears. A matched comparison of the yield of two smears collected using the standard (*spot-morning*) and the front-loading (*spot-Xspot*) approaches stratified by country is shown in Table 3. Again, the proportion of cases missed by the *spot-morning* and the *spot-spot* smears is not statistically different.

Fifty-one (25%) of the 206 patients in Nepal and 72 (29%) of the 250 patients in Yemen had a positive TB culture. Thirty-nine and 37 of the 51 culture-positive patients in Nepal were identified by the standard and the front-loaded schemes, resulting in a sensitivity of 76% [95%CI 63%–87%] and 73% [59%–83%], respectively. In Yemen, 51 and 52 of the 72 culture-positive patients were identified by the standard and front-loaded schemes, respectively, (sensitivity 71% [60%–80%] and 72% [61%–82%]). There were no statistical differences between the sensitivities of the standard and front-loaded approaches.

The sensitivity of two smears collected as *spot-morning* would be 69% (35 of 51 culture positive patients) in Nepal and 71% (51 of 72) in Yemen. In comparison, the sensitivity of the two smears collected as *spot-Xspot* would be 67% (34 of 51) in Nepal and 67% (48 of 72) in Yemen. These differences are not statistically significant (*P* > .4 for both).

## 4. Discussion

Much progress has been made in recent years in the development of new diagnostics for TB. However, few of the newer diagnostic technologies are suitable for use outside of reference laboratories in the public health services of LMICs. TB control in resource-poor, high-prevalence settings, therefore, will continue to rely upon sputum smear microscopy until frontline services gain access to the new technologies. Tuberculosis affects disproportionately indigent populations who seek health services in areas with limited resources [11], and the costs incurred by patients can be prohibitively high, even when services are provided free of charge [12]. Individuals need to attend health facilities on several occasions to see a clinician, submit sputum specimens, receive results, and be put on treatment, and these visits may incur loss of earnings and require repeated travel, purchase of food, and accommodation [13]. A significant proportion of people undergoing investigations for TB drop-out of the smear diagnostic pathway, and these patients are more likely to be the poor. Although few studies report the proportion of patients who drop out during the diagnosis of TB in LMIC, 13% of TB suspects in India [14], 15% and 37% of smear-positive patients in rural [8], and urban Malawi [15] dropout of the process, and unusually, the diagnostic dropout rate can be as high as 95% [16]. Failing to complete the diagnosis, therefore, is a major obstacle to access treatment in these settings [8].

There is increasing interest in equity in health services, as typified by the interim report of the WHO Commission on Social Determinants of Health. Primary health care, once again, plays a central role in WHO's current agenda, and the development of equitable diagnostic services is, therefore, paramount to access treatment [17]. The investigation of suspected PTB in many low-prevalence countries is based on the examination of serial *morning* specimens because these specimens have been associated with a significant additional yield of patients. The proportion of patients who drop out in these settings, however, is insignificant, and the *spot-morning-spot* scheme was developed in the 1950s and 60s in response to the need to reduce the number of visits in high-prevalence areas, where patients often abandoned the diagnostic process. Although it was widely accepted that overnight specimens were more likely to contain more bacilli, it was also acknowledged that in less favourable circumstances, it was more practical to obtain specimens at the time the patient was attending the service [18]. Two of the morning specimens were replaced by spot specimens, and shorter schemes that required a reduced number of visits were developed. Although this was a remarkable improvement, the continued need for multiple visits was still a hindrance for many patients.

It is now recognised that the high bacillary threshold for defining a smear as positive and the requirement to obtain at least one confirmatory smear have unnecessarily reduced the sensitivity of the test in the detection of smear positive cases. These requirements also resulted in many laboratory services being overwhelmed and leaving insufficient time for the examination of smears. The recent WHO policy changes reduce the smear microscopy thresholds [9] and the minimum number of specimens to be examined. These changes will reduce workload, particularly important in areas, where skilled human resources are limited, potentially increasing case detection through allowing more time to examine the smears [10]. These policy changes might be associated with larger gains in case detection if the timing of sputum specimen collection and examination were more convenient for patients, particularly poor patients, and help to reduce drop-out.

This study shows that the *spot-spot-morning* and *spot-morning-spot* schemes have similar yields. This indicates that front-loaded TB diagnostic services (whether based on the examination of two or three specimens) are feasible and would not be associated with significantly less yield than the equivalent standard approach. Both two-smear strategies would miss about 3%–5% of the patients identified by the three-specimen strategy, as suggested by a previous systematic review [5, 19]. These losses are likely to be compensated by increased quality of microscopy and lower drop-out rates of patients. In addition, programmes where ≥10% of patients fail to return for the second day of diagnosis would identify similar numbers using 2 smears the first day of consultation than 2 or 3 smears collected over two or more days. Countries adopting two-smear schemes, therefore, may consider collecting and examining the specimens in a single day to shorten the time required for diagnosis.

The development of diagnostic approaches that are responsive to the needs of the population may be feasible, and larger studies are urgently required to validate the findings of this study under operational conditions. If the findings of this study are confirmed, smear microscopy services should be front loaded in the interests of equity and improved TB control.

## 5. Summary

The diagnosis of tuberculosis in high-burden settings relies on sputum smear microscopy and requires multiple patients' visits to the health facilities. This approach could be improved if most specimens were collected the first day of consultation. This study reports the smear microscopy findings of 923 adults with chronic cough participating in four cross-sectional surveysin Ethiopia, Nepal, Nigeria, and Yemen. Sputum specimens were collected as spot-morning-spot plus one additional specimen one hour after the first spot (X-spot). The yield of two (spot-Xspot or spot-morning) or three (spot-morning-spot or spot-X-spot-morning) specimens was compared. 216 patients had ≥ one positive smear. Of these, 210 (97%) were identified by the spot-morning-spot, and 210 (97%) were identified by the spot-Xspot-morning specimens. Spot-morning identified 203 and spot-Xspot specimens 200 patients, respectively, (*P* > .1). The time, number of visits and patients' costs to complete smear microscopy could be reduced by frontloading the collection of sputum specimens.

## Figures and Tables

**Table 1:** Incremental yield of serial smears collected as spot-morning-spot or spot-Xspot-morning specimens.

Country	N*	≥1 pos. smear,	First spot pos.	Standard approach			Front-loaded approach		
		*N* (%)	*N* (%)	Incremental yield, *N* (%)		*N* (%) detected	Incremental yield, *N* (%)		*N* (%) detected
				Morning	Second spot		Xspot	Morning
Ethiopia	243	52 (21)	45 (87)	4 (8)	2 (4)	51(98)	4 (8)	3 (6)	52 (100)
Nepal	206	55 (25)	39 (71)	7 (13)	5 (9)	51 (93)	5 (9)	6 (11)	50 (91)
Nigeria	224	48 (21)	45 (94)	3 (6)	0	48 (100)	2 (4)	1 (2)	48 (100)
Yemen	250	61 (24)	52 (85)	8 (13)	0	60 (98)	7 (12)	1 (2)	60 (98)

All	923	216 (23)	181 (84)	22 (10)	7 (3)	210 (97)	18 (8)	11 (5)	210 (97)

*N = number; pos = positive; Xspot = Extra spot collected one hour after the first spot sputum specimen.

**Table 2:** Yield of the third smear for samples collected using the standard (spot-morning-spot) and the front-loading (spot-spot-morning) approaches by country. 62 patients (15 from Yemen, 44 from Nepal, and 3 from Ethiopia) were excluded as they did not submit the third specimen.

Standard approach	Front-loading approach			P*
*Yemen* (*N* = 235)	Pos on 1st two smears	Only 3rd smear pos	All negative	.14
Pos on 1st two smears	56	4	0	
Only 3rd smear pos	0	0	0	
All negative	0	0	175	

*Nepal* (*N* = 162)				.76
Pos on 1st two smears	40	6	0	
Pos on the 3rd smear	3	0	5	
Negative in all	3	0	105	

*Nigeria* (*N* = 224)				.61
Pos on 1st two smears	47	1	0	
Pos on the 3rd smear	0	0	0	
Negative in all	0	0	176	

*Ethiopia* (*N* = 240)				.55
Pos on 1st two smears	46	3	0	
Pos on the 3rd smear	2	0	0	
Negative in all	1	0	188	

*All sites* (*N* = 861)				.58
Pos on 1st two smears	189	14	0	
Pos on the 3rd smear	5	0	5	
Negative in all	4	0	644	

*Stuart-MaxWell test, (Marginal Heterogeneity).

**Table 3:** Yield of two smears collected using the standard (spot-morning) and the frontloading (spot-Xspot) approaches by country.

Standard approach	Frontloading approach			P*
*Yemen*	Positive	Negative	All	.18
Positive	56	4	60	
Negative	1	189	190	
All	57	193	250	

*Nepal*				.53
Positive	40	6	46	
Negative	4	156	160	
All	44	162	206	

*Nigeria*				.32
Positive	47	1	48	
Negative	0	176	176	
All	47	177	224	

*Ethiopia*				1.0
Positive	46	3	49	
Negative	3	191	194	
All	49	194	243	

*All sites*				.58
Positive	189	14	203	
Negative	8	712	720	
All	197	726	923	

*McNemar test.
